# A Measure of the Impact on Real-Time Patient Care of Evidence-based Medicine Logs

**DOI:** 10.5811/westjem.18082

**Published:** 2024-06-20

**Authors:** Jeffrey B. Brown, Ajay K. Varadhan, Jacob R. Albers, Shreyas Kudrimoti, Estelle Cervantes, Phillip Sgobba, Dawn M. Yenser, Bryan G. Kane

**Affiliations:** Lehigh Valley Health Network, University of South Florida Morsani College of Medicine, Department of Emergency and Hospital Medicine, Allentown, Pennsylvania

## Abstract

**Introduction:**

Evidence-based medicine (EBM) is a critical skill for physicians, and EBM competency has been shown to increase implementation of best medical practices, reduce medical errors, and increase patient-centered care. Like any skill, EBM must be practiced, receiving iterative feedback to improve learners’ comprehension. Having residents document patient interactions in logbooks to allow for residency program review, feedback, and documentation of competency has been previously described as a best practice within emergency medicine (EM) to document practice-based learning (PBL) competency. Quantifying how residents use the information they query, locate, evaluate, and apply while providing direct patient care can measure the efficacy of EBM education and provide insight into more efficient ways of providing medical care.

**Methods:**

Practice-based learning logs were surveys created to record resident EBM activity on-shift and were placed into our residency management software program. Residents were required to submit 3–5 surveys of EBM activity performed during a 28-day rotation during which additional information was sought. This study included all PBL logs completed by EM residents from June 1, 2013–May 11, 2020. Using qualitative methodology, a codebook was created to analyze residents’ free-text responses to the prompt: “Based on your research, would you have done anything differently?” The codebook was designed to generate a three-digit code conveying the effect of the researched information on the patient about whom the log was written, as well as whether the information would affect future patient care and whether these decisions were based on scientific evidence.

**Results:**

A total of 10,574 logs were included for primary analysis. In total, 1,977 (18.7%) logs indicated that the evidence acquired through research would affect future patient care. Of these, 392 (3.7%) explicitly stated that the EBM activity conducted as part of our project led to real-time changes in patient care in the ED and would change future management of patients as well.

**Conclusion:**

We present a proof of concept that PBL log activity can lead to integration of evidence-based medicine into real-time patient care. While a convenience sample, our cohort recorded evidence of both lifelong learning and application to patient care.

Population Health Research Capsule What do we already know about this issue?

*Quantifying how residents use information retrieved from scientific evidence is an important subject in need of further investigation.*

What was the research question?

*Does evidence found by residents impact their current or future clinical practices?*

What was the major finding of the study?

*18.7% of logs showed that the EBM search would affect future patient care, and 3.7% stated it changed ED patient care in real time.*

How does this improve population health?

*More effective quantification of evidence-based clinical practice changes will allow instructors to identify educational gaps and close them.*

## INTRODUCTION

Medical diagnostics and treatments are constantly changing, making it difficult for physicians to stay current with the care they provide. Evidence-based medicine (EBM), which is the process of researching and applying new medical information, falls under the broader educational category of practice-based learning (PBL).^1^^,^^2^ Evidence-based medicine is a critical skill for physicians, and EBM competency increases implementation of best medical practices, reduces medical errors, and improves patient-centered care^3^^,^^4^. EBM is frequently used to generate policies and guidelines to improve the quality of care delivered.^5^ Thus, it is crucial to learn and apply EBM skills throughout medical training. Like any skill, EBM must be practiced, receiving iterative feedback. Prior literature has demonstrated the value of the use of logbooks to document resident progression toward competency.^6^

The Accreditation Council on Graduate Medical Education (ACGME) has mandated that residency programs monitor resident performance in multiple areas, including EBM, within the PBL competency.^1^ Resident competence is measured by observable and measurable ACGME Milestone behaviors.^7^ Specific approaches to these requirements are not externally defined; rather they are left to both the program director and the appointed program evaluation committee (PEC).^1^ Having residents document patient interactions in logbooks to allow for residency program review, feedback, and documentation of competency has been previously described as best practice within EM to document PBL competency.^8^ Prior study of EBM has demonstrated that postgraduate experience and gender both impact the learning needs of residents.^9^

Evidence-based medicine consists of four key steps: asking an answerable question; efficiently searching for evidence; appraising the evidence for reliability; and applying that evidence.^10^ A 2010 survey of EM program directors and faculty reported that the most important EBM skillsets developed by residents were the ability to appraise the reliability of evidence they find and apply research findings to patient care.^8^ Resident surveys can be used to record how each individual approaches EBM, although this approach has the same inherent limitations of all survey studies. The Fresno test of EBM is a standardized means of assessment and feedback on the topic.^11^ The test takes around 40 minutes to complete and 12 minutes to grade.^12^ However, none of these EBM education studies measure its impact on direct patient care, despite Kirkpatrick’s hierarchy placing impact on patient care at the top of the educational evaluation pyramid.^13^

Prior research has demonstrated that real-time EBM may lead to implementation of best care practices.^14^ Quantifying how residents use the information they query, locate, evaluate, and apply when providing direct patient care can measure the efficacy of EBM education and yield insight into more efficient ways to provide medical care.^15^ Our purpose in this study was to review residents’ PBL patient logs as a measure of EBM activity among residents and to determine the direct impact of this EBM activity on both current and future patient care.

## METHODS

This was an institutional review board-approved retrospective review of self-reported learning conducted at an ACGME-approved postgraduate year (PGY) 1–4 EM residency program, which trains approximately 14 residents per year. The program is located at an independent academic center within an integrated healthcare network. The EBM curriculum at this institution was taught primarily within interactive journal clubs based on PGY and was supplemented with didactics that involved real-time audience response questions, in accordance with best practices.^9^ The core journal club (attended by PGY-1 and -2 residents) measured educational efficacy with the Fresno test of EBM and had topics based on its content.^11^ Residents took the Fresno test of EBM and received feedback on their performance on that instrument during protected time in grand rounds in May or June at the end of PGY-2. Senior journal club (attended by PGY-3 and -4 residents) focused on critical appraisal, knowledge translation, and implementation science. The senior journal clubs contained separate learning goals and assignments. Depending on grand round didactic scheduling and faculty availability, the audience response system questions and interactive discussions occurred both in large group (all residents PGY 1–4) and small groups-based settings (PGY 1, 2 in one room, and PGY 3, 4 in another). Both journal clubs used a recurring 12-month curriculum containing standardized EBM teaching articles paired with topical clinical exercises (Table 1). Core journal club materials were assigned to the PGY-1 and -2 residents and senior materials to the PGY-3 and -4 residents.

**Table 1.** The evidence-based medicine curriculum administered to residents over the course of a year.

Month/Topic (Fresno question)	Evidence-based medicine core teaching article	Senior and supplemental materials
July PICO question (1a, 1b)	Guyatt G, Meade MO, Agoritsas T, et al. (2015). What is the question? In Guyatt G, Rennie D, Meade MO, Cook DJ (Eds.), *Users’ Guides to the Medical Literature: A Manual for Evidence-Based Clinical Practice, 3rd ed*. (1–9) New York, NY: McGraw Hill.	Senior journal club: Resident as teacher. Barrett NF, Gopal B. Using the five microskills with different learning preferences. *Fam Med*. 2008;40(8):543–5.
August Hierarchy and locations of evidence (2, 3, 11, 12)	Bhandari M, Giannoudis PV. Evidence-based medicine: What it is and what it is not. *Injury*. 2006;37(4):302–6.	Senior journal club: Knowledge translation Lang ES, Wyer PC, Haynes RB. Knowledge translation: closing the evidence-to-practice gap. *Ann Emerg Med*. 2007;49(3):355–63.
September Search strategies (2, 3, 4)	McKibbon A, Wyer P, Jaeschke, R, et al. (2002). Finding the evidence. In Guyatt G, Rennie D, Meade MO, & Cook DJ (Eds.), *Users’ Guides to the Medical Literature: A Manual for Evidence-Based Clinical Practice 2* *nd* *ed.* (29–58). New York, NY: McGraw-Hill Medical.	Senior journal club: Implementation science de Wit K, Curran J, Thoma B, et al. Review of implementation strategies to change healthcare provider behaviour in the emergency department. *CJEM*. 2018;20(3):453–60.
October External validity (5)	Rothwell PM. External validity of randomised controlled trials: “to whom do the results of this trial apply?”. *Lancet*. 2005;365(9453):82–93.	Supplemental articles on 2x2 grids: Loong TW. Understanding sensitivity and specificity with the right side of the brain [published correction appears in BMJ. 2003 Nov 1;327(7422):1043]. *BMJ*. 2003;327(7417):716–9. Gallagher EJ. Evidence-based emergency medicine/editorial. The problem with sensitivity and specificity. *Ann Emerg Med*. 2003;42(2):298–303.
November Likelihood ratios (8)	Hayden SR, Brown MD. Likelihood ratio: a powerful tool for incorporating the results of a diagnostic test into clinical decisionmaking. *Ann Emerg Med*. 1999;33(5):575–80.	Supplemental lecture: Bayesian logic. This additional lecture outside of journal club introduces basic 2x2 grid concepts and extends them into how to use EBM on shift to achieve clinical diagnosis. Slawson DC, Shaughnessy AF. Teaching information mastery: the case of baby Jeff and the importance of Bayes’ theorem. *Fam Med*. 2002;34(2):140–2.
December Number needed to treat (9)	Cordell WH. Number needed to treat (NNT). *Ann Emerg Med*. 1999;33(4):433–6.	No supplemental materials given this month. Both resident groups used core materials indicated immediately to the left.
January Significance (7)	Singer AJ, Thode HC Jr, Hollander JE. Research fundamentals: selection and development of clinical outcome measures. *Acad Emerg Med*. 2000;7(4):397–401.	Senior journal club: Sources of critical appraisal. These sources are used for the critical appraisal forms throughout the year. Forms include *Annals of Emergency Medicine* (https://www.annemergmed.com/content/ebemform) and the Centre for Evidence Based Medicine, which has a collection in multiple languages (https://www.cebm.ox.ac.uk/resources/ebm-tools/critical-appraisal-tools).
February Critical appraisal: diagnostics	Schranz DA, Dunn MA. Evidence-based medicine, part 3. An introduction to critical appraisal of articles on diagnosis. *J Am Osteopath Assoc*. 2007;107(8):304–9.	Supplemental article on methodology: Thompson CB, Panacek EA. Research study designs: experimental and quasi-experimental. *Air Med J*. 2006;25(6):242–6.
March Critical appraisal: therapeutics	Cardarelli R, Virgilio RF, Taylor L. Evidence-based medicine, part 2. An introduction to critical appraisal of articles on therapy. *J Am Osteopath Assoc*. 2007;107(8):299–303.	Supplemental lecture: Review of Methodology This lecture is given outside of journal club to review and reinforce the hierarchy of evidence and the internal validity of articles.
April Communication	Montori VM, Devereaux PJ, Straus S, et al. (2002). Advanced topics in moving from evidence to action: decision making and the patient. In Guyatt G, Rennie D, Meade MO, & Cook DJ (Eds.), *Users’ Guides to the Medical Literature: A Manual for Evidence-Based Clinical Practice 2* *nd* *ed.* (643–61). New York, NY: McGraw-Hill Medical.	No supplemental materials given this month. Both resident groups used core materials indicated immediately to the left.
May (OPEN EBM topic, research day)	Note: Topic determined by EBM Track Residents Note 2: Question 6 on the Fresno (internal validity) is reviewed in the appraisal of articles in each journal club.	No supplemental materials given this month. Both resident groups used core materials indicated immediately to the left.
June Confidence intervals (10)	Young KD, Lewis RJ. What is confidence? Part 1: The use and interpretation of confidence intervals. *Ann Emerg Med*. 1997;30(3):307–10.	No supplemental materials given this month. Both resident groups used core materials indicated immediately to the left.

*PICO*, population, intervention, control, and outcomes; *EBM*, evidence-based medicine.

Supplemental materials were used by all residents during two one-hour EBM didactics held outside journal club. In the fall, the lecture covered 2x2 grids/likelihood ratios/Bayesian logic. In the spring, the lecture reviewed commonly used research methodologies. The clinical content of the December and June journal clubs was rapid abstract review from the most recent meetings of the American College of Emergency Physicians (December) and the Society for Academic Emergency Medicine (June). The clinical content for the remaining journal clubs was selected from recent literature to highlight the core topic being taught that month.

After review and approval by the PEC, residents were required to document EBM activity in the program’s procedure recorder. These records, referred to as PBL logs, were developed from patient follow-up logs disseminated by the Council of Residency Directors in Emergency Medicine (CORD).^7^^,^^16^ Table 2 demonstrates the elements of the PBL logs’ associated expectations, and areas of feedback by PGY. These logs were from a convenience sample of EBM activity and were not a record of all patients seen. One faculty member reviewed every log, and each resident was provided with individualized feedback from the same faculty member.

**Table 2.** Practice-based learning logs and expectations, stratified by postgraduate year. The top row indicates practice-based learning log categories, while rows 2–4 indicates the expected capabilities of residents.

PGY	Clinical question	Clinical question answer	Method of obtaining information	Based on your research, would you have done anything differently?
1	PICO question, search strategy		Identify source of information and verify reliability	
2	Search strategy	Evidence found		Identify significance of the information
3 + 4		Evidence found		Critical appraisal of information reliability, application of information to practice

*PGY*, postgraduate year; *PICO,* population, intervention, control, and outcomes.

Residents were required to create their PBL logs during rotations in the emergency department (ED). These logs were subsequently placed into our residency management software program (New Innovations Inc, Uniontown, OH). Residents were required to submit surveys of EBM activity performed during 28-day EM rotations. The number required per rotation by the PEC varied between the academic years included in this study from a high of five at the beginning of the cohort down to three at the end. The annual number of EM rotations varied by PGY, from six (PGY 1) to eight (PGY 4). The number of residents per class at the beginning of the cohort was 13, and the complement increased to 16 during the study.

We included all PBL logs completed by EM residents from June 1, 2013–May 11, 2020. Records were anonymized to PGY year and gender, in accordance with Hadley et al.^9^ No other identifiers were included in this study. Using qualitative methodology described by MacQueen, we created a codebook to analyze residents’ free-text responses to the prompt: “Based on your research, would you have done anything differently?”^17^ The goal of the codebook was to categorize and quantify the effect of each log on a resident’s patient care. Each log was assigned a three-digit code based on the answer to three questions. The first digit of the code corresponded to the answer to the question, “Did the research affect the care of the current patient about which the log was written?” The second digit represented participant answers to the question, “Will information researched change the future care of patients?” Lastly, the third digit represented the answer to the question, “Were the changes described in digit two in concordance with the research they found?” Digits were assigned to answer each of these questions (Table 3).

**Table 3.** Practice-based learning log codebook. The answers to these three questions were coded to generate a three-digit number describing the impact a resident’s research had on their performance.

	Digit 1: Did research affect care?	Digit 2: Will research affect care in the future?	Digit 3: Is the change in future care based on the researched evidence?
Incomplete log	9	N/A	N/A
Duplicate log	8	N/A	N/A
Yes	1	1	1
No	2	2	2
Maybe	3	3	3
Action influenced by outside force (eg, attending physician preference, state protocol, did not have access to medication, etc)	4	4	4
Undecipherable answer	5	5	5
Insufficient or conflicting data	N/A	N/A	7

Logs were coded by three individuals with a single over-riding adjudicator. All individuals involved in coding logs met to code the first 200 logs together to create a consensus for grading and met throughout the entirety of the project to review logs with unclear coding. Inter-rater reliability was not formally measured. We conducted subgroup analysis based on PGY and resident gender via the chi-square test to assess for differences in log coding. If the resident did not specify their gender or PGY, we excluded the log from the respective subgroup analysis.

## RESULTS

A total of 11,145 logs were entered during the study period. These logs were submitted by 137 residents, of whom 48 (37%) were female. We excluded a total of 571 logs from analysis: 298 were incomplete, and 273 were duplicates. After these exclusions, 10,574 logs answered the prompt, “Based on your research, would you have done anything differently?” and were included in primary analysis (Figure).

**Figure.**
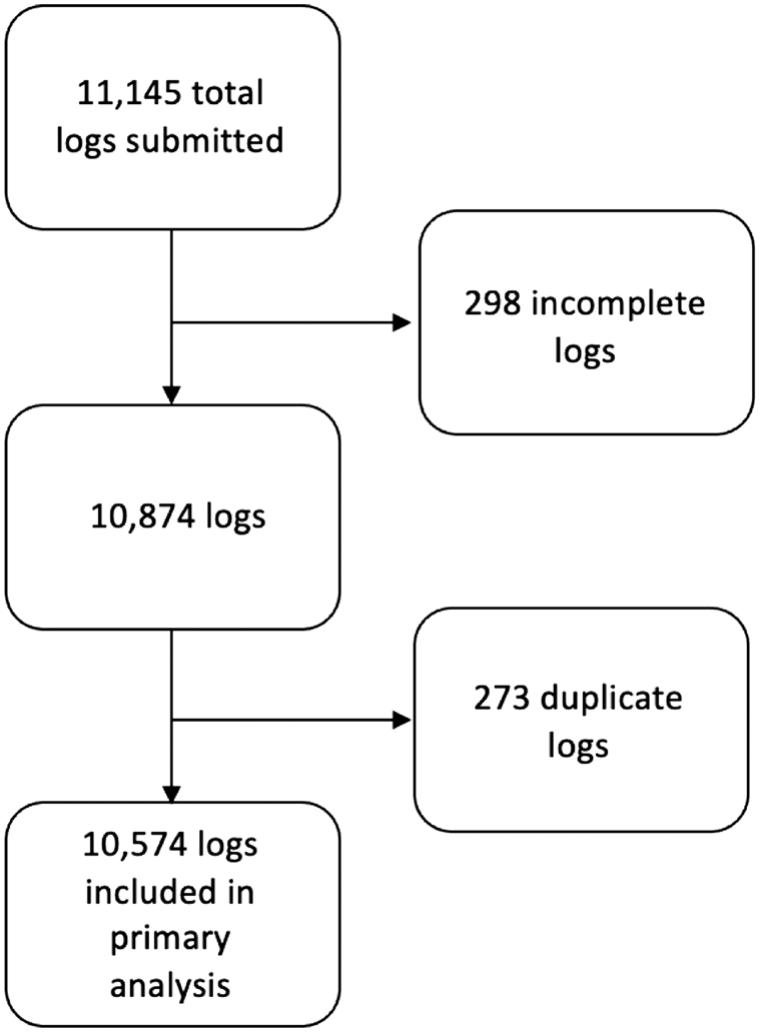
Practice-based learning log study inclusion criteria and selection process.

The five most common log codes accounted for approximately 85.4% (n = 9,034) of the total logs. The most common log code was 231 in 3,343 logs (31.6%), which signified self-directed learning without application of knowledge to the current patient and without specifying application of knowledge to future patients. The second most common code was 331 in 2,263 logs (21.4%), which similarly recorded self-directed learning without specification of application of knowledge to the current or future patients. Research confirming residents’ plans of care was coded as 221 and totaled 1,319 logs (21.4%). The next highest count was 211in 1,062 logs (10.0%), which represented logs that did not change the care of the corresponding patient but reportedly would change the care of future patients. The code 131 accounted for 1,047 (9.9%) logs, which changed the care of the corresponding patient and may or may not change future care of patients.

The most impactful logs were those that specifically stated that the research conducted would change future management of patients (eg, coded as 111, 211, 311, 411). In total, 1,977 logs (18.7%) indicated the evidence acquired through research would affect future patient care. Of these, 392 (3.7% of entire study sample) explicitly stated that the EBM activity conducted as part of our project led to real-time changes in patient care in the ED and would change future management of patients as well. A full list of the 10 most common codes can be found in Table 4.

**Table 4.** The 10 most commonly reported practice-based learning log counts, stratified by postgraduate year.

Code	Total (%)	PGY1 (%)	PGY2 (%)	PGY3 (%)	PGY4 (%)
231	3,343 (31.6)	880 (26.3)	877 (26.2)	679 (20.3)	907 (27.1)
331	2,263 (21.4)	450 (19.9)	522 (23.1)	512 (22.6)	779 (34.4)
221	1,319 (12.5)	278 (21.1)	311 (23.6)	298 (22.6)	432 (32.8)
211	1,062 (10.0)	348 (32.8)	249 (23.4)	202 (19.0)	263 (24.8)
131	1,047 (9.9)	246 (23.5)	221 (21.1)	230 (22.0)	350 (33.4)
311	443 (4.2)	134 (30.2)	114 (25.7)	82 (18.5)	113 (25.5)
111	392 (3.7)	97 (24.7)	92 (23.5)	92 (23.5)	111 (28.3)
431	265 (2.5)	57 (21.5)	67 (25.3)	59 (22.3)	82 (30.9)
227	97 (0.9)	21 (21.6)	22 (22.7)	20 (20.6)	34 (35.1)
411	80 (0.8)	23 (28.8)	22 (27.5)	12 (15.0)	23 (28.8)
Other	263 (2.5)	53 (20.2)	68 (25.9)	61 (23.2)	81 (30.8)

*PGY*, postgraduate year.

Postgraduate year subgroup analysis found first-year residents recorded the most logs (602, 23.3%) that indicated research would lead to a future change in patient management (all codes including 1 as the second digit). There was a significant difference in these logs between PGY (*P* < 0.001), and a trend was seen where increasing PGY was associated with decreased chance of a log changing future patient care. The number of logs indicating future care changes increased in PGY 4, potentially due to dedicated feedback received on their PBL logs. There was no significant difference in the number of logs that recorded both real-time change and future management change between PGY (*P* = 0.70). Subgroup analysis of resident gender found no significant difference in current or future patient care resulting from evidence found. While not meeting the study’s significance criterion of α < 0.05, more males indicated real-time and future care change in their logs (254), as compared to their female counterparts (106, *P* = 0.05). Subgroup analysis can be seen in Table 5.

**Table 5.** Distribution of practice-based learning logs and their effects, stratified by gender and postgraduate year.

	Total number of logs (%)	Future care change (eg, 111, 211, etc) (%)	*P*-value	Real-time and future care change (eg, 111 only) (%)	*P*-value
Gender					
Female	3,505 (34.3)	617 (33.1)	0.27	106 (29.4)	0.05
Male	6,705 (65.7)	1,246 (66.9)		254 (70.6)	
Total	10,210 (100.0)	1,863 (100.0)		360 (100.0)	
Postgraduate year					
1	2,587 (24.5)	602 (30.5)	<0.001	97 (24.7)	0.70
2	2,565 (24.3)	477 (24.1)		92 (23.5)	
3	2,247 (21.3)	388 (19.6)		92 (23.5)	
4	3,175 (30.0)	510 (28.3)		111 (28.3)	
Total	10,574 (100.0)	1,977 (100.0)		392 (100.0)	

## DISCUSSION

Evidence-based medicine forms the cornerstone of modern clinical practice, and effectively teaching residents to conduct their own EBM information acquisition and appraisal is paramount in graduate medical education. While both the ACGME and CORD require that EBM be taught throughout residency, there is little data assessing the impact and relative benefit to residents of EBM search methods. Our analysis included 10,574 PBL logs from 137 residents across eight academic years. Our results showed that 18.7% of logs indicated that residents acquired new evidence-based medical information and applied that knowledge in real-time to change the current or future care of their patients. These positive educational logs were found more often in the logs of PGY-1 and -4 residents than in those of PGY-2 and -3 residents. This observation may be related to background searches leading to new knowledge (PGY 1) and the ability to critically appraise (PGY 4). Changing learning styles in residency has been shown to be linked to the number of hours worked, and in our training program the PGY-2 and -3 residents have more intense rotations than in PGY-1.^18^ Another contributing factor to the increase seen in PGY-4 residents could be the dedicated feedback on this component of the PBL log, which is provided to PGY-3 and -4 residents (Table 2).

Evidence-based medicine, like other residency procedures, is a learned skill that must be practiced.^19^ Logs are consistently used across residency programs to track progress of traditional skills; however, there is limited literature describing how to track EBM skill progression.^20^ Some elements of teaching, particularly providing feedback to learners, are acknowledged as beneficial to skill development.^21^ The approach described here, using a program’s traditional log and faculty feedback to assess EBM like other procedural competencies, has been described as a best practice.^8^^,^^21^ In addition to the discussion and demonstration of EBM skills including appraisal that occurred in journal club, individual feedback was given by a single faculty member (as shown in Table 2) of all logs in the clinical competency committee meetings and in residents’ semi-annual evaluations. Minimal literature exists wherein the effect of resident EBM activity on patient care was measured. Friedman et al did investigate this question; however, the study was criticized for not offering a way for residents to improve their appraisal skills.^22^^,^^23^

By conducting EBM learning in an interactive journal club setting, supplemented with didactics that involved real-time audience response questions, students were able to engage with their instructors and receive more comprehensive feedback on their methods and the implications of their findings. In addition, we used an electronic tool that directly linked EBM resources to the electronic health record to ease access during rotations. These expanded functionalities also allowed us to monitor the implementation of residents’ newly acquired knowledge more directly. By specifically asking how the activity impacted patient care, the PBL logs enabled the residency program to gather information on ACGME Phase 3 Outcomes data.^24^

A key finding of our study was the high number of logs that demonstrated real-time learning. While only 18.7% of logs explicitly stated a real-time or planned future change in patient care, over 65% of logs reported that research led to information learned. This is an important finding, as self-directed learning is a growing academic topic. The most recent iteration of the ACGME Milestones for EM included EBM within two PBL categories.^7^ Practice-based learning 1 (Evidence-based and Informed Practice) has at levels 1, 2, 3, and 4 behaviors that can be measured with the PBL logs. Further, PBL 2 (Reflective Practice and Commitment to Personal Growth) has as behavioral anchors the ability to self-identify gaps and determine ways to close them. The PBL logs, by measuring self-directed learning, contribute to measuring these behaviors in an effective way. As the Liaison Committee on Medical Education has made “self-directed learning” (Standard 6.3) a mandated part of undergraduate medical education, the approach to EBM described here can effectively extend and measure that behavior.^25^ There are currently no existing resources to do so; thus, our study model provides a novel way for residency programs to track self-directed learning of EBM via PBL logs.

## LIMITATIONS

Despite our large study population, our study had multiple limitations. The EBM curriculum offered and described in Table 1 may differ from that offered at other academic sites. Therefore, the way that residents used information may not be representative of EM residents at other institutions. The recorded EBM activity was a requirement for our residents, including a minimum number of PBL logs using peer-reviewed, published sources of medical information. This cohort, therefore, had the general limitations of convenience sampling, as well as the possibility of bias, as EBM activities were not as thoroughly documented as procedural attempts. To that end, the logs presented likely represent only a fraction of the EBM activity performed during the study period as only a limited number of logs (3–5) were required for each 28-day EM block. Finally, our qualitative methodology required the interpretation and categorization of EBM logs, which introduced the possibility for interpretation bias in our results. However, the large number of logs, the use of multiple research team members with a single arbiter, and the coding schema developed minimized these concerns.

## CONCLUSION

We present a proof of concept that practice-based learning log activity can lead to integration of evidence-based medicine into real-time patient care. Additionally, we provide a framework for qualitative measurement of EBM research and application skills among learners. Our cohort recorded evidence of both lifelong learning and application to patient care. This approach can easily be generalized to other EM residencies to allow for both monitoring of resident PBL competency and ACGME reporting.

## Supplementary Information




