# Supplementary material for: A Measure of the Impact on Real-Time Patient Care of Evidence-based Medicine Logs
Source: West J Emerg Med. 2024 Jun 20;25(4):565–73. doi: 10.5811/westjem.18082 (PMC11254145; doi:10.5811/westjem.18082)
Supplement: Supplementary file 1 [file wjem-25-565-s001.docx]

| **ACGME Milestone** | **Competency level** | **Behavior to be demonstrated** |
| --- | --- | --- |
| Practice-Based Learning and Improvement 1: Evidence-Based and Informed Practice | Level 1 | Demonstrates how to access and use available evidence |
|  | Level 2 | Articulates the clinical questions that are necessary to guide evidence-based care |
|  | Level 3 | Locates and applies the best available evidence, integrating it with patient preference, to the care of complex patients |
|  | Level 4 | Critically appraises and applies evidence even in the face of uncertainty and of conflicting evidence to guide care that is tailored to the individual patient |
|  | Level 5 | Coaches others to critically appraise and apply evidence for complex patients, and/or participates in the development of guidelines |
| Practice-Based Learning and Improvement 2: Reflective Practice and Commitment to Personal Growth | Level 1 | Demonstrates an openness to performance data (feedback and other input) |
|  | Level 2 | Demonstrates an openness to performance data and uses it to develop personal and professional goals  Identifies the factors that contribute to the gap(s) between expectations and actual performance |
|  | Level 3 | Seeks and accepts performance data for developing personal and professional goals  Analyzes and reflects upon the factors that contribute to gap(s) between expectations and actual performance |
|  | Level 4 | Using performance data, continually improves and measures the effectiveness of one’s personal and professional goals  Analyzes, reflects on, and institutes behavioral change(s) to narrow the gap(s) between expectations and actual performance |
|  | Level 5 | Acts as a role model for the development of personal and professional goals  Coaches others on reflective practice |

Appendix 2: ACGME requirements for demonstrable PBL behaviors in Emergency Medicine residency. Requirements were gathered from the 2021 ACGME Emergency Medicine Milestones.
